# Supplementary figures and images for: Maternal and perinatal mortality and morbidity of uterine rupture and its association with prolonged duration of operation in Ethiopia: A systematic review and meta-analysis
Source: PLoS One. 2021 Apr 22;16(4):e0245977. doi: 10.1371/journal.pone.0245977 (PMC8062067; doi:10.1371/journal.pone.0245977)

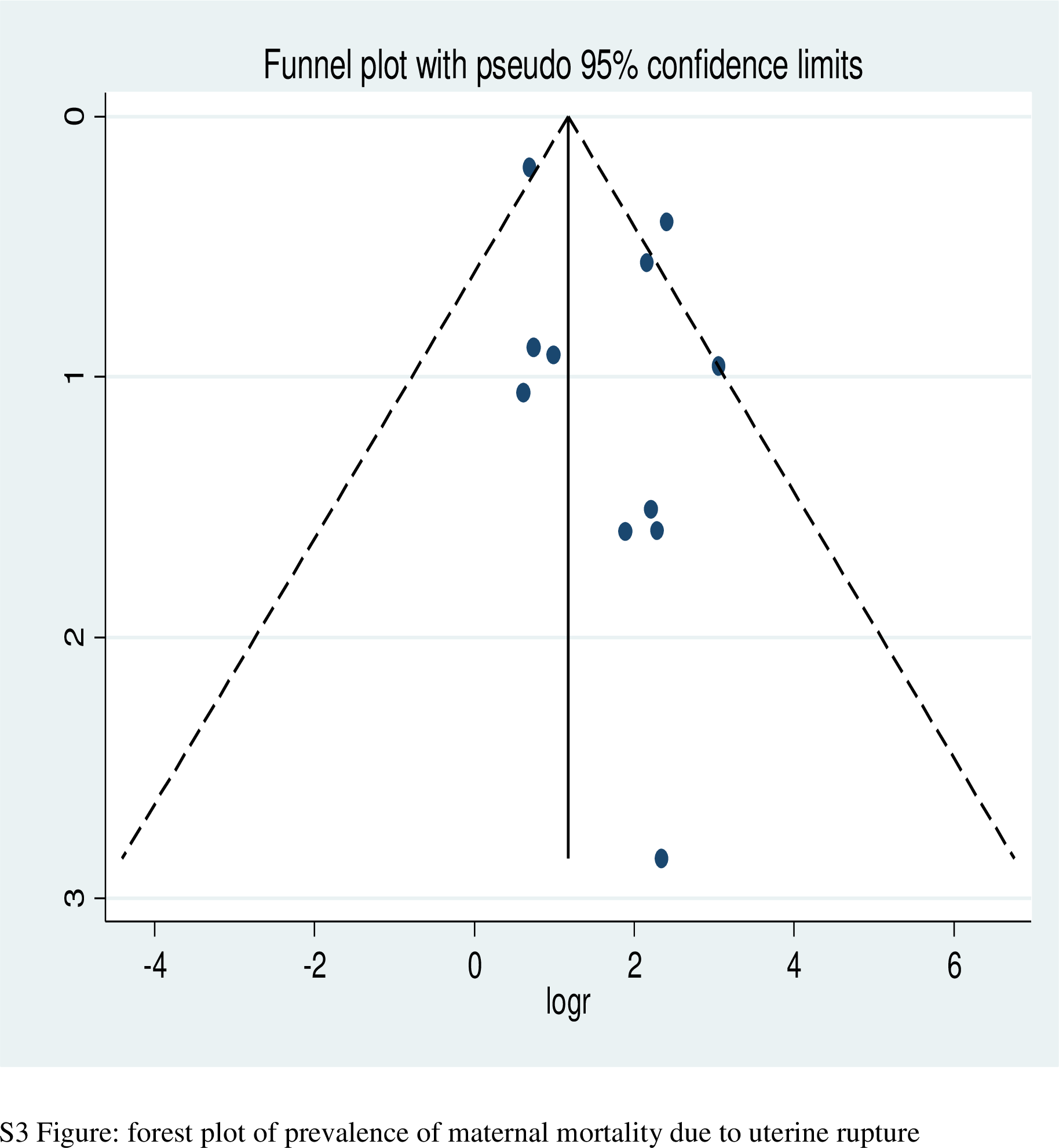

Supplement: S1 Fig — (TIF) [file pone.0245977.s002.tif]

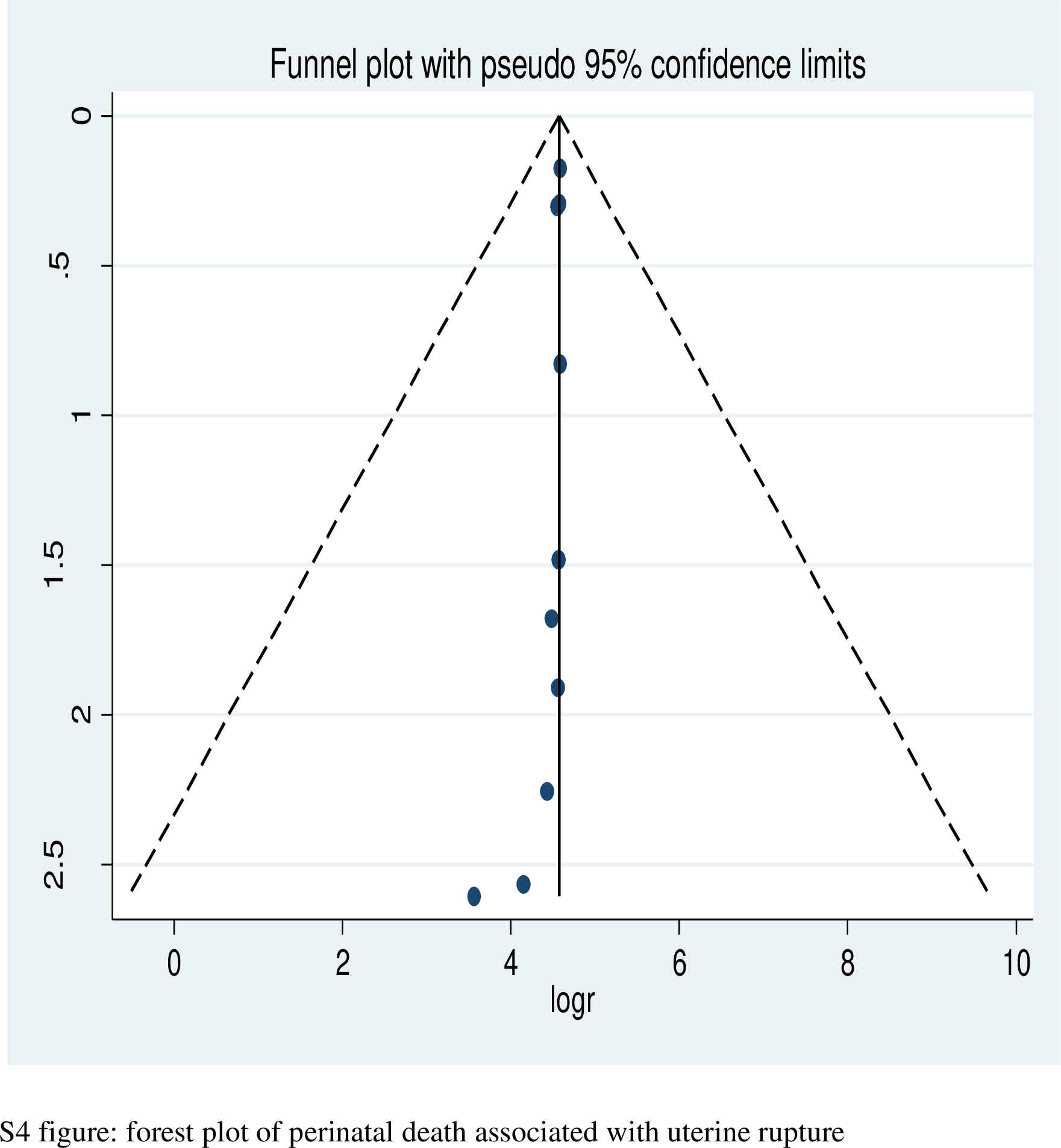

Supplement: S2 Fig — (TIF) [file pone.0245977.s003.tif]
