# Supplementary material for: Maternal and perinatal mortality and morbidity of uterine rupture and its association with prolonged duration of operation in Ethiopia: A systematic review and meta-analysis
Source: PLoS One. 2021 Apr 22;16(4):e0245977. doi: 10.1371/journal.pone.0245977 (PMC8062067; doi:10.1371/journal.pone.0245977)
Supplement: S1 Table — (DOCX) [file pone.0245977.s004.docx]

S2 Table: PubMed search string of maternal and perinatal mortality and morbidity of uterine rupture

| #1 | "maternal mortality" [MeSH Terms] OR "maternal mortality" [Text Word] |
| --- | --- |
| #2 | "maternal complications"[MeSH Terms] OR “maternal complications" [Text Word] |
| #3 | (((("Puerperal Infection"[Mesh]) OR ("Puerperal Infection"[text word]) OR (("Shock"[Mesh] OR "Shock" [Text Word]) OR ("Shock, Hemorrhagic"[Mesh]) OR "Shock, Septic"[Mesh]) OR "Anemia"[Mesh] OR "Anemia"[Text Word])) |
| # 4 | "perinatal mortality"[MeSH Terms] OR "perinatal death"[MeSH Terms] OR perinatal mortality [Text Word] |
| # 5 | uterine rupture"[MeSH Terms] OR (("uterine"[All Fields] AND ("rupture"[All Fields]) OR "uterine rupture"[All Fields]) |
| #6 | Ethiopia [Mesh] OR Ethiopia [tiab] |
| # 7 | (#1 AND # 5) |
| # 8 | ((#2 OR #3) AND # 5) |
| # 9 | (# 4 AND # 5) |
| #10 | (((# 7 AND # 8) AND # 9)) AND 6) |
