# Supplementary material for: Maternal and perinatal mortality and morbidity of uterine rupture and its association with prolonged duration of operation in Ethiopia: A systematic review and meta-analysis
Source: PLoS One. 2021 Apr 22;16(4):e0245977. doi: 10.1371/journal.pone.0245977 (PMC8062067; doi:10.1371/journal.pone.0245977)
Supplement: Attachment — Submitted filename: Comments on MM and PM Ethiopia.docx [file pone.0245977.s005.docx]

Manuscript #: PONE-D-20-12283

**Title: Maternal and perinatal mortality and morbidity of uterine rupture and its association with prolonged duration of operation in Ethiopia: a systematic review and meta-analysis**

This study looked at uterine rupture operation time as it relates to maternal and perinatal mortality and morbidity. Using a systematic review and meta-analysis gives this study the potential to present one of the highest levels of evidence.

One cannot help but commend the efforts of the authors in carrying out this study. My comments on the various sections are as written below. On a general note the whole of the discussion section needs to be reviewed due to a lot of grammatical errors which clouds the intended messages.

Summary

The summary of the results was concise but no mention was made of the predictors of perinatal morbidity. In your conclusion in the summary you mentioned that improving the duration of surgery with WHO standards was recommended to reduce maternal and perinatal mortality and morbidity; could the skill of the surgeon be important since this has a direct bearing on the quality and duration of surgery?

Background

In the second paragraph you wrote “A multicounty survey showed that uterine rupture associated with adverse maternal outcomes: maternal near-miss, maternal mortality, fresh stillbirth and perinatal death” Do you mean “A multicounty survey showed that uterine rupture is associated with adverse maternal outcomes: maternal near-miss, maternal mortality, fresh stillbirth and perinatal death” ? Kindly clarify.

Methods

Under: Eligibility criteria and study selection; you wrote “All published studies in the English language up to the end of our searching (30/6/2019) ……” When was the beginning of the research?

Under: Outcome measurement; it was written “Maternal morbidities: women who have uterine rupture who acquire had developed at least of one of the following conditions: ….” Kindly clarify.

Under: Quality assessment and data collection: you wrote: “Then, two authors (MD and HA) independently assessed …..” This was also mentioned under Eligibility criteria and study selection. I am not sure if you need to mention the initials of these authors within the text of the methods. Would it have sufficed to have mentioned that “some selected authors amongst us did ……”? There is a section in the forms given by journal editors that gives you the opportunity to write out the specific contribution of authors.

Results

In the first paragraph under: Study identification and characteristics of included studies it was written: “An international electronic database such as PubMed, Cochrane Library, Google Scholar, and African Journals Online databases were used for search strategy.” Is this relevant here, as this was already mentioned under Study design and search strategy?

In Table 1: Characteristics of included studies in Ethiopia: Is it possible to write the years of the studies serially?

Under: Association of prolonged duration of the operation and maternal morbidity; did you consider type of rupture as being a factor?

Discussion

In the first paragraph it was written: “In this setting, the high burden of obstructed labour (25) and complications resulting in metabolic disturbances; sepsis and shock, which subsequently end up with maternal death.” This sentence is not clear. Also “A study done in Debre Markos also supports the evidence that those women died due to uterine rupture, 37% mothers died from shock, …” Do you mean “A study done in Debre Markos also supports the evidence that those women died due to uterine rupture, 37% of mothers died from shock, …”?

This sentence ” Thus, to reduce the high burden of maternal mortality, prevention of associated morbidities as haemorrhage, shock and sepsis, the leading cause of maternal mortality, can be accomplished through the timely delivery by instrumental / CS for prolonged labor before resulting of uterine rupture and reduction of anemia, usually accomplished prenatally with iron-folic acid and vitamin supplementations and pharmacological stimulation should be improved.” Is not clear. Can it be written as “Thus, to reduce the high burden of maternal mortality due to uterine rupture, prevention of morbidities like haemorrhage, shock and sepsis which are the leading causes, can be accomplished through timely delivery by vacuum, forceps or caesarean section for prolonged labour with appropriate indications. Prenatally, anaemia can be prevented by Iron, Folic acid and vitamin supplementation. Protocols for induction and stimulation of labour also needs improvement.”?

This sentence: “Despite, thus finding is similar with studies done in Nigeria (27) 6.6% of maternal mortality associated with uterine rupture and it was lower than the Senegal and Mali’s report, 12% (28) and Sudan,14.3% ..” is not clear. Do you mean “Despite our findings, it is similar with studies….?

This sentence: “This might due to the high proportion of women in the area…” do you mean “This might be due to the high proportion of women in the area…”?

Kindly explain this sentence: “The findings of this meta-analysis also found that the highest prevalence of maternal mortality associated with uterine rupture has occurred in SNNPR region and the lowest was in Tigray region. Are SNNPR and Tigray regions rural, semi urban or urban areas?

Rephrase this sentence: “This explained due to the fact that those women who had who have a uterine rupture in Ethiopia might be less likely get skilled birth earlier within the golden time due to the poor decisions about when to seek care at the point of childbirth (10, 11, 31), results delay in getting care and uterine rupture, increases the risk of uterine rupture associated fetal mortality.”

Kindly rephrase this sentence: “Despite, it was the first review and meta-analysis in Ethiopia, and the included studies show the causal relationship, the findings of this review interpreted based on some limitation.”

TO THE EDITOR: On a general note the whole of the discussion section needs to be reviewed due to a lot of grammatical errors which clouds the intended messages.

TO THE EDITOR: On a general note the whole of the discussion section needs to be reviewed due to a lot of grammatical errors which clouds the intended messages. This article can be considered for publication if these corrections are made. Thank you.

PROF. HAJARATU UMAR SULAYMAN.

13^TH^ JULY 2020.
