# Supplementary material for: Maternal and perinatal mortality and morbidity of uterine rupture and its association with prolonged duration of operation in Ethiopia: A systematic review and meta-analysis
Source: PLoS One. 2021 Apr 22;16(4):e0245977. doi: 10.1371/journal.pone.0245977 (PMC8062067; doi:10.1371/journal.pone.0245977)
Supplement: Attachment — Submitted filename: Response to reviewers.docx [file pone.0245977.s006.docx]

**Dear Editors and reviewers of Plos One**

We would like to extend our deepest appreciation for your scholarly comments and devoting your time to review our manuscript entitled “Maternal and perinatal mortality and morbidity of uterine rupture and its association with prolonged duration of operation in Ethiopia: a systematic review and meta-analysis”. Uterine rupture is a global public problem that causes maternal and perinatal morbidity and mortality, which can be prevented through estimating pooled data at national level for interventions. Even though, the prevalence is inconsistent across the country. Therefore, this systematic review and meta-analysis estimates the pooled maternal and perinatal mortality and morbidity and the effect of prolonged duration of labour on maternal morbidity in Ethiopia.

Dear reviewer, there has been a major revision of the whole structure of the manuscript (Abstract, introduction, methods, results, discussion and conclusions) mainly with a correction of grammar errors. The journal requirements supposed by you have been included as search strategy has included as supplementary file and the whole structure of the manuscript has been revised to be easy for readers. We hope now the manuscript is clear and more acceptable than its previous version. We tried to reduce the overlap with previous publications and we also cite the sources in the methods section. We have tried to present the response for each reviewer according to your comment what to supposed to do so. For this, here we have given our responses to each of the concerns you raised, highlighted by red color. Again, we would like to remind our strongest gratitude for your effort for the improvement of this manuscript and the response for each the points were addressed in the response to reviewers’ section.

**Regards**

**Reviewers comment and response**

**Reviewer one**

\# 1. **Overall**: On a general note the whole of the discussion section needs to be reviewed due to a lot of grammatical errors which clouds the intended messages.

**Thank you for the highly valuable comments**

The whole of the discussion section has been revised and grammatical errors was corrected which to increase clarity the intended messages to the readers.

\# 2. ***Summary***: The summary of the results was concise but no mention was made of the predictors of perinatal morbidity. In your conclusion in the summary you mentioned that improving the duration of surgery with WHO standards was recommended to reduce maternal and perinatal mortality and morbidity; could the skill of the surgeon be important since this has a direct bearing on the quality and duration of surgery?

**We accepted it and thank you for highly scholarly comments.**

Unfortunately, we estimate the pooled effect of prolonged duration of surgery on maternal morbidities only due to the absence of data in the included studies. Thus, in regard to the absence of predictor on perinatal mortality we have explicitly discussed in the introductions and limitations section.

Yes, the skill of the surgeon be important for the improvement of the duration of surgery with WHO standards since this has a direct bearing on the quality and duration of surgery. Moreover, the duration of operation also depends on dependent on the type and extent of rupture, hemodynamic status of the mother, desire for future fertility, presence of gross infection and experience of the surgeon.

#3. **Background**: In the second paragraph you wrote “A multicounty survey showed that uterine rupture associated with adverse maternal outcomes: maternal near-miss, maternal mortality, fresh stillbirth and perinatal death” Do you mean “A multicounty survey showed that uterine rupture is associated with adverse maternal outcomes: maternal near-miss, maternal mortality, fresh stillbirth and perinatal death”?

**Accepted and revisions have made in the background.**

**#4. Methods**

4.1. Under: Eligibility criteria and study selection; you wrote “All published studies in the English language up to the end of our searching (30/6/2019) ” When was the beginning of the research?

**Thanks, the valuable comment.** The beginning of this research was done after checking of the absence of published and registered protocol PROSPERO 13/02/2019 and systematic search was started after 27/03/2019. In the meantime, due to connection problem, academic activities and participating in research and community service prolonged the accomplishment of our manuscript.

4.2. Under: Outcome measurement; it was written “Maternal morbidities: women who have uterine rupture who acquire had developed at least of one of the following conditions: ….” Kindly clarify.

**Accepted and revised in the outcome measurement section.**

**“**Maternal morbidity is a defined when a woman who had uterine rupture develop at least one of the following conditions like sepsis, post-partum hemorrhage, anemia, relaparatomy...**”**

4.3. Under: Quality assessment and data collection: you wrote: “Then, two authors (MD and HA) independently assessed ….” This was also mentioned under Eligibility criteria and study selection. I am not sure if you need to mention the initials of these authors within the text of the methods. Would it have sufficed to have mentioned that “some selected authors amongst us did ……”? There is a section in the forms given by journal editors that gives you the opportunity to write out the specific contribution of authors. **Accepted and removed from this section**.

#5. Results

5.1. In the first paragraph under: Study identification and characteristics of included studies it was written: “An international electronic database such as PubMed, Cochrane Library, Google Scholar, and African Journals Online databases were used for search strategy.” Is this relevant here, as this was already mentioned under Study design and search strategy?

**Accepted and removed from the results section.**

Hence, it is not relevant here, as this was already mentioned under Study design and search strategy.

5.2. In Table 1: Characteristics of included studies in Ethiopia: Is it possible to write the years of the studies serially?

**Valuable comment and the studies were writing serially.**

5.3. Under: Association of prolonged duration of the operation and maternal morbidity; did you consider type of rupture as being a factor?

**Highly valuable and future studies should consider it**

Unfortunately, absence of data on the primary included studies the type of uterine rupture was not considered. Despite, type of uterine rupture affects the type of surgery and the duration of operation. Overall, regardless of type of rupture to save the lives of the women and the neonate improving the duration of operations is recommended. Duration of operation in this case is used as an average for either of complete of incomplete uterine rupture based on previous research from the original studies.

Thus, we have mentioned in the discussions and limitations to consider it in future studies.

\# 2. The aim and title need revising. The title seems to suggest that the study aims to find out the association between morbidity/mortality and duration of operation. However, the results section deliberates more on analysis of maternal and perinatal outcomes of the 11 studies and very little about the effect of duration of operation. This makes it difficult to decipher the key message from the manuscript. Is the study aiming at evaluating compliance to the 22 items in the WHO checklist? Is it assessing the association between duration of operation and maternal/perinatal outcomes? How will this help improve maternal and perinatal care in Ethiopia?

\#**6. Discussion**

6.1. In the first paragraph it was written: “In this setting, the high burden of obstructed labour (25) and complications resulting in metabolic disturbances; sepsis and shock, which subsequently end up with maternal death.” This sentence is not clear. Also “A study done in Debre Markos also supports the evidence that those women died due to uterine rupture, 37% mothers died from shock, …” Do you mean “A study done in Debre Markos also supports the evidence that those women died due to uterine rupture, 37% of mothers died from shock, …”?

**Accepted and revision had made.**

“A study conducted in Debre Markos also showed that 37% of women with uterine rupture died from shock, 25% from severe anemia and 19% died from septic shock.”

6.2. This sentence ” Thus, to reduce the high burden of maternal mortality, prevention of associated morbidities as haemorrhage, shock and sepsis, the leading cause of maternal mortality, can be accomplished through the timely delivery by instrumental / CS for prolonged labor before resulting of uterine rupture and reduction of anemia, usually accomplished prenatally with iron-folic acid and vitamin supplementations and pharmacological stimulation should be improved.” Is not clear. Can it be written as “Thus, to reduce the high burden of maternal mortality due to uterine rupture, prevention of morbidities like haemorrhage, shock and sepsis which are the leading causes, can be accomplished through timely delivery by vacuum, forceps or caesarean section for prolonged labour with appropriate indications. Prenatally, anaemia can be prevented by Iron, Folic acid and vitamin supplementation. Protocols for induction and stimulation of labour also needs improvement.”?

**Accepted and revised based on what you supposed to do so.**

6.3. This sentence: “Despite, thus finding is similar with studies done in Nigeria (27) 6.6% of maternal mortality associated with uterine rupture and it was lower than the Senegal and Mali’s report, 12% (28) and Sudan,14.3% ..” is not clear. Do you mean “Despite our findings, it is similar with studies….? This sentence: “This might due to the high proportion of women in the area…” do you mean “This might be due to the high proportion of women in the area…”?

**Accepted and revised based on comments.**

The sentence was revised as “ The findings of this study was similar with a study done in Nigeria (27), 6.6% of maternal mortality associated with uterine rupture, but lower than the Senegal and Mali’s report (12%) (28) and a study from Sudan (14.3%)”.

The sentence was writing as “This might be due to the high proportion of women in the area”.

6.4. Kindly explain this sentence: “The findings of this meta-analysis also found that the highest prevalence of maternal mortality associated with uterine rupture has occurred in SNNPR region and the lowest was in Tigray region. Are SNNPR and Tigray regions rural, semi urban or urban areas?

**Thank you for your valuable comment**

The region distribution in Ethiopia is mix of rural, semi urban or urban areas including SNNPR and Tigray regions. Based on the 2016 ETHIOPIAN HOUSEHOLD CONSUMPTION EXPENDITURE (HCE) SURVEY statistical report the country has four domains rural, major urban centers, medium towns and small size towns. Except Addis Ababa administrative city (city doesn’t have a rural part) all regions have four domains. Thus, SNNPR and Tigray regions has rural, semi urban or urban areas. Specifically, there was 35 rural woredas or districts, urban inhabitants’ number 844,040 or 20 % of the population in based on 2007 census in Tigray region.

6.5. Rephrase this sentence: “This explained due to the fact that those women who had who have a uterine rupture in Ethiopia might be less likely get skilled birth earlier within the golden time due to the poor decisions about when to seek care at the point of childbirth (10, 11, 31), results delay in getting care and uterine rupture, increases the risk of uterine rupture associated fetal mortality.”

**Accepted and revisions had made.**

6.6. Kindly rephrase this sentence: “Despite, it was the first review and meta-analysis in Ethiopia, and the included studies show the causal relationship, the findings of this review interpreted based on some limitation.”

**Thank you for this highly scholarly comment and the revisions had made.**

**Reviewer two**

**#1**. **Aim and title**: This need revising. The title seems to suggest that the study aims to find out the association between morbidity/mortality and duration of operation. However, the results section deliberates more on analysis of maternal and perinatal outcomes of the 11 studies and very little about the effect of duration of operation. This makes it difficult to decipher the key message from the manuscript. Is the study aiming at evaluating compliance to the 22 items in the WHO checklist? Is it assessing the association between duration of operation and maternal/perinatal outcomes? How will this help improve maternal and perinatal care in Ethiopia?

**Thank you for highly valuable comments**

We have tried to address it in the introduction clearly and the limitations sections what you supposed to do. The results section deliberates more on analysis of maternal and perinatal outcomes was due to three or more outcomes were investigated on the area. The meta-analysis can’t assess the effect of prolonged duration of operation maternal and perinatal mortality due to absence of data on the included studies. But, the effect of prolonged duration of operation on maternal morbidities was estimated. Thus, we have recommended future prospective studies should address the gap here. Hence, any studies have its limitations.

It is well mentioned and narrated in the discussions section on the effect of duration of operation on maternal and perinatal outcomes; the duration of operation is one of the plans of save lives with safe surgery. In case of uterine rupture with obstructed labour the neonate might be asphyxiated and in distress and the women might be on shock either hypovolemic or septic shock, as the duration of operation improves, the neonate and the women might get the care immediately before end up of detah. As example, the neonate might get life support care as resuscitation and CPAP, cardiopulmonary air pressure or resuscitation, and oxygen therapy.

In regard to the compliance to the 22 items in the WHO checklist, it was mentioned in our discussion sections as implications / area of improvements and proper use of the recommendations. Hence, it is means of improving operation or surgical care.

#2. **Design**: The study identifies more with a literature review and meta-analysis of data than a systematic review. Within the PECO framework chosen, the exposure suggested is “duration of operation” however performing an obstetric operation involves other items that may confound the duration measured. This includes the nature and extend of the uterine rupture, the type of anesthesia used, level of health care, availability of equipment and consumables. A statement acknowledging possibility of such confounders would be helpful.

**Thank you for the highly valuable scholarly comments and revised in the discussions**

Straight forward, the nature and extend of the uterine rupture, the type of anesthesia used, level of health care, availability of equipment and consumables. Moreover, the duration of operation is dependent on the type and extent of rupture, hemodynamic status of the mother, desire for future fertility, presence of gross infection and experience of the surgeon. This is mentioned it in our discussions section.

**#4.** Key terms used: Terms such as morbidity, shock and anaemia may need some more clarification to improve understanding. For example, shock is reported as a leading cause of morbidity. It remains to clarify how morbidity was defined in this study and in the studies included in the review. It will also be helpful to know when complications such as anaemia were diagnosed. How long after the uterine rupture? A mention of if complications were outlined as immediate and long term in the studies and if they were presented separately.

**Thank you for a valuable comment and revision was made.**

Morbidity, shock and anaemia was defined in this study and based on what the included primary studies define. In regard to the maternal morbidities, sepsis and anemia; a woman was be declared she has the event or the outcome if she is diagnosed and treated as recorded in the original included studies. Hence, shock and anemia are the leading causes of maternal mortality due to uterine rupture, immediate diagnosis and management is needed. Postpartum anemia was defined as low blood hemoglobin concentration below ≤ 10 g/dl or hematocrit level less than ≤30% at 24 hours postpartum and severe anemia: if the below 9 g/dl or hematocrit level less than 27% at 24 hours postpartum. A separate estimate of the complications (as immediate and long term) were not done due absence of data in the studies.

**#5.** Causal effect: The paragraph just before the conclusion section asserts that this meta-analysis has shown a causal relationship. This is a strong statement that needs clarity into how it was reached. Similarly, the statement…”and an improvement of the health care system is our country due to the commitment of the government to reduce maternal mortality with increased qualified staffs” may need rephrasing or evidence for the same.

**Accepted and responded**

**The** causal relationship is due to the nature of included studies, in which cohort studies were used.

Based on the Ethiopian Demographic Heath survey (EDHS) 2016 report, there was an increase in antenatal care and institutional deliveries and decrease of problems in Ethiopia, are the most indicators for the improvements of healthcare. Example,

1- The proportion of women age 15-49 in Ethiopia who received antenatal care (ANC) from a skilled provider has increased from 27% in 2000 to 34% in 2011, and 62% in 2016. Thirty-two percent of women had at least four ANC visits during their last pregnancy.
2- Institutional deliveries have increased from 5% in 2000 to 10% in 2011, and 26% in 2016. During the same period, home deliveries decreased from 95% in 2000 to 90% in 2011, and 73% in 2016.
3- Problems in accessing health care: The proportion of women age 15-49 who report having at least one of the specified problems in accessing health care decreased from 96% in 2005, to 94% in 2011, and 70% in 2016.

The evidence was cited in the discussions.

\# 6. Grammar and typos need attention throughout the document. No page numbers indicated.

**Accepted and revised**

The grammar and typos have extensively edited and revised throughout the document and page number was indicated.

Thank you for your scholarly constructive comments.

Regards
