# Supplementary material for: Maternal and perinatal mortality and morbidity of uterine rupture and its association with prolonged duration of operation in Ethiopia: A systematic review and meta-analysis
Source: PLoS One. 2021 Apr 22;16(4):e0245977. doi: 10.1371/journal.pone.0245977 (PMC8062067; doi:10.1371/journal.pone.0245977)
Supplement: Attachment — Submitted filename: Response to Reviewer .docx [file pone.0245977.s007.docx]

**Dear editors of PLOS ONE**

We would like to extend our deepest appreciation for your scholarly comments and devoting your time to review our manuscript entitled “Maternal and perinatal mortality and morbidity of uterine rupture and its association with prolonged duration of operation in Ethiopia: a systematic review and meta-analysis”. Uterine rupture is a global public problem that causes maternal and perinatal morbidity and mortality, which can be prevented through estimating pooled data at national level for interventions. Even though, the prevalence is inconsistent across the country. Therefore, this systematic review and meta-analysis estimates the pooled maternal and perinatal mortality and morbidity and the effect of prolonged duration of labour on maternal morbidity in Ethiopia.

Dear reviewer, there has been a minor revision of the whole structure of the manuscript mainly with a correction of editing errors to be easy for readers. We hope now the manuscript is clear and more acceptable than its previous version. We have tried to present the response for each reviewer according to your comment what to supposed to do so. For this, here we have given our responses to each of the concerns you raised, highlighted by red color. Again, we would like to remind our strongest gratitude for your effort for the improvement of this manuscript and the response for each the points were addressed in the response to reviewers’ section.

**Regards**

Reviewer #2: Thanks for addressing many of the comments given during the first review. However, I am afraid, there is more to do in terms of use of technical terms and grammatical correctness.

**Thank you for the highly valuable and scholarly comments**

#1. In the methods section (Page 4), I don't think you need to present the basic information about Ethiopia. You could safely delete the second and third sentences.

Response: Highly valuable comment, I completely agree what you supposed and revision was made.

#2. It will be helpful for the conclusion to be clear whether increased duration of operation is associated with increased severity or number of complications. This is not yet clear.

**Response: valuable and responses.** We have done analysis based on the objectives we have plan to do or defined in the outcome measurement section, the pooled effect of prolonged duration of operation on maternal morbidity than the counterparts than the severity or number of complications**.** Clinically that is not such surprising that the severity of complications is increased among prolonged duration, if the presence of maternal morbidity associated with prolonged duration of labour is assured. To talk as a scholar than that of the clinical point of view, it was interesting to estimate the severity or the number of complications due to prolonged duration. When we said severity, it needs categorization like mild, moderate and severe or another category. But we only do the pooled estimate base on the available evidences from the original articles. Unfortunately, we didn’t get any evidence in regard to the severity or the number of complications encounter associated with prolonged duration of operation. We have got the composite outcome of maternal morbidity or complications. Hence, we have done a pooled estimate on the effect of prolonged duration of operation on maternal morbidities or complications based on what we have plan to do so. Thus, identifying the level of severity or number of complications of prolonged duration of operation is the area of future research. We have put it on the limitations section.

#3. There is inconsistent use of the terms stillbirth, perinatal deaths, neonatal deaths eg page 3. Please check and revise. A stillbirth is also a perinatal death. The fourth statement on page 5: ... "Perinatal death of uterine rupture is defined as death of the fetus after 28 weeks of birth to the first week of neonatal or early neonatal period from uterine rupture, its complications or management".

**Response:** accepted and revision was made what you suggested to do so.

#4. Page 7: Not mentioned how you ended up with 11 studies from 16, although the information is available on the PRISMA diagram.

**Response: valuable** and the information on how to reach 11 studies from the total 16 was included.

#5. Some final grammatical considerations: Examples: Page 3: Third paragraph... complication instead of complications., Page 5: ... a hospital based published studies. "a" is not required.

Please double check throughout the manuscript.

Response: accepted and revision was made.

Thank you for the highly valuable comments and we tried to check throughout the manuscript.

Reviewer 3

1. Abstract

a. As a whole includes 368 words while in the journal regulation should not exceed 300!!!!

**Response**: accepted and the abstract was revised based on the journal regulations.

b. The background is too long and away from your objectives i.e The adverse outcomes of uterine rupture were highly variable and inconclusive across different studies in the country is recorded in the background and your objectives was not to find out the true adverse outcomes !!!! What were your adverse out comes???

**Response: Thank you for your highly valuable comments and modification was made on it.**

But what we have to implies that there was variability in regard to the proportion of outcomes instead of the true outcome or not. Thus, we have tried to list out the common adverse outcomes of uterine rupture in the background and also the analysis. Our intention is to estimate the pooled maternal and perinal mortality and morbidity due to uterine rupture. Hence, there was no national level data. Thus, to get a national level data for an input for policy makers we do this meta-analysis.

c. Specifying the effect of increased maternal and perinatal outcomes after UR just by (prolonged duration of operation in Ethiopia) is not justifiable!!! As you have to elude other causes for increasing this rate for instance delayed of presentation to the hospital! The woman was in collapse state at time of admission that u failed to resuscitate her and the woman was died not because of the prolonged operation time? The women had heart disease with RU!!!! The operation was prolonged because she had rupture bladder with the RU!!! may be

**Response**: **thank you for the** **highly valuable comments.**

We have tried to estimate maternal morbidities associated with prolonged duration of operation based on the available evidences from the original studies. As you have suggested that other cofounders like delay to referral or seeking care, reach to the hospital, delay in getting the treatment and other medical disorders like health disease might affect the outcome. Unfortunately, we have narrated in the limitations section that we didn’t estimate the pooled effect of prolonged duration of operation on maternal and perinatal mortality. We only assess the pooled effect of prolonged duration of operation on maternal morbidity. Even though the morbidity even may matter on women with having antenatal medical disorders like heart disease and others. We understand that the duration of operation might be prolonged in case of bladder rupture with uterine rupture, in case there is an increasing bleeding and reduce the time to get other services in the recovery room which might affect the outcome. We can’t do this because due to absence of available data on the original articles and no such population with this outcome. Upto our point of view, it needs other study objective or problem statement, hence uterine rupture with bladder rupture is rare case that might needs Poisson regression. But the included studies were cross-sectional and case control studies doesn’t consider the charactertics of populations in the count data. Dear reviewer studies have its own limitations and we have put it in the limitation section.

d. Conclusion: Uterine rupture was associated with maternal morbidity and prolonged duration of the operation was found to be associated with maternal morbidities. Again u have to clarify this statement in the methodology.

**Response**: valuable and it was stated in the methodology section.

e. The Preferred Reporting Items for Systematic Reviews and Meta-Analyses (PRISMA) mentioned once in abstract, no need to add the abbreviation here.

**Response**: accepted and revision was made, it already deleted from the abstract.

f. Therefore, birth preparedness and complication readiness plan, early referral and improving the duration of operation according to the World Health Organization standards are recommended: that is true all these factors involves to increase morbidity and mortality but the problem u haven’t identify these factors in your methodology or result to compare it with the duration of operation.

**Response**: Thank you for the valuable comments. We haven’t identified birth preparedness and complication readiness plan and early referral as a factor in the methods and results section. However, what we have conclude is that based on the implications or the discussions section highlighted that absence of birth preparedness and complication readiness plan and delay in referral are the possible justifications for the high burden of maternal and perianal mortality/ morbidity due to uterine rupture. Thus, we have suggested this based on the imperative findings what was done before that what we have found. Thus, if you suggest to remove if from the conclusions section, we will remove it in the final version. But all other co-authors and I preferred to be putted as it. But what you supposed to do is an area of future research.

g. to reduce maternal morbidity and mortality and stillbirth among women with uterine rupture: Please unify your results regarding what do you looked for in the article in relation to fetal or neonatal out comes .Was it just stillbirth rate or perinatal outcome? , or admission to NICU?

**Response**: we have revised the conclusions based on what we have found in the results section. It was perinatal outcome and we have revised it based on comments.

2. Introduction:

a. WHO developed a checklist consisting of 22-items to be used at critical perioperative moments of induction, incision, and before leaving the operating room : These check lists should be identified in detail in methods section.

**Response: accepted and the checklist was identified in the methods section.**

Outcome measurement:

1. Change the title to : Outcome measurements and definitions

**Response: accepted and revised based on what you suggest to do so.**

2. Second line: Uterine rupture is a partial…… uterine rupture was defined as ….

**Response: revision was made**

3. U have to define all your variables here , Maternal mortality, Morbidity , perinatal death, stillbirth, sepsis ,severe anemia Fistula (which type , shock, …..and all should have references

**Response: accepted and correction was made based on your suggestion.**

4. Maternal morbidities of uterine rupture include sepsis, shock, severe anemia and fistula: Maternal morbidities after uterine rupture include much more morbidities which were not included in this study!!! u have to clarify u haven’t identify them and why u have only recorded (sepsis, fistula, shock and severe anemia).

Response: Thank you for the valuable comments. you are right what you supposed to do so. There are many morbidities associated with uterine rupture which are not included in the separate analysis. We only included sepsis, fistula, shock and severe anemia because the morbidities were reported many times from the original articles that fitted for meta-analysis and will give an input for national policy than other nonincluded morbidities. Unfortunately, we estimate the overall pooled prevalence of maternal morbidity of uterine rupture. Separate analysis like pooled estimate of sepsis, fistula, shock and severe anemia were supplementary or second objectives. Even, it was possible to include those other morbidities as narrative description, but most authors suggest to put only the pooled estimate morbidities in this paper. If you suggest that narrating in descriptive manner other remained morbidities is a such important, we welcome your suggestion.

5. How long women after operation were followed up to confirm Fistula and sepsis?

**Response: Valuable comments**

Fistula and sepsis declared if the women are diagnosed and treated as recorded in the original included studies. Hence, sepsis is the leading causes of maternal mortality due to uterine rupture, immediate diagnosis and management is needed. While, prevention of fistula and diagnosis of fistula should be done preoperatively, intraoperatively and postoperatively. Hence, fistula is occupied with uterine rupture with obstructed labour. Thus, an integrating team of the in the operating room like catheterization and strict follow-up from operation till discharge weather the mother having morbidities like sepsis and fistula.

6. Are there any women died before the operation was conducted for them??? And did they included in the analysis or excluded?

Result: Screening of their title and abstracts. finally, a total of 16 full-text papers were assessed for eligibility based on the prior inclusion and exclusion criteria. Therefore, a total of 11 studies were included in the final meta-analysis: why 11 out of 16?? please identify the cause.

Response: we putted in the results section and in the PRISMA check list.

**Thank you for your consideration.**

**Regards**
